# The Carniolan Honeybee from Slovenia—A Complete and Annotated Mitochondrial Genome with Comparisons to Closely Related *Apis mellifera* Subspecies

**DOI:** 10.3390/insects13050403

**Published:** 2022-04-22

**Authors:** Ajda Moškrič, Andraž Marinč, Polonca Ferk, Brane Leskošek, Mai-Britt Mosbech, Ignas Bunikis, Olga Vinnere Pettersson, Lucile Soler, Janez Prešern

**Affiliations:** 1 Animal Production Department, Agricultural Institute of Slovenia, Hacquetova ulica 17, SI-1000 Ljubljana, Slovenia; andraz.marinc@kis.si (A.M.); janez.presern@kis.si (J.P.); 2 Faculty of Medicine, Institute for Biostatistics and Medical Informatics/Centre ELIXIR-SI, University of Ljubljana, Vrazov trg 2, SI-1000 Ljubljana, Slovenia; polonca.ferk@mf.uni-lj.si (P.F.); brane.leskosek@mf.uni-lj.si (B.L.); 3 Uppsala Genome Center, Science for Life Laboratory, Department of Immunology, Genetics and Pathology, Uppsala University, BMC, Box 815, 752 37 Uppsala, Sweden; mai-britt.mosbech@igp.uu.se (M.-B.M.); ignas.bunikis@scilifelab.uu.se (I.B.); olga.pettersson@igp.uu.se (O.V.P.); 4 Department of Medical Biochemistry and Microbiology (IMBIM), Uppsala University, National Bioinformatics Infrastructure Sweden (NBIS), Science for Life Laboratory, 751 24 Uppsala, Sweden; lucile.soler@nbis.se

**Keywords:** western honeybee, *Apis mellifera carnica*, mitochondrial genome, phylogeny, C lineage, annotation, whole genome sequencing, protection, conservation

## Abstract

**Simple Summary:**

The western honeybee, *Apis mellifera*, is a globally distributed bee species with many recognised subspecies, one of which is *Apis mellifera carnica*, the Carniolan honeybee. *Apis m. carnica* is native to southern Central Europe and parts of the Balkans, with the *locus classicus* in Slovenia. It is also widely popular with beekeepers in parts of Central and Northern Europe and other parts of the world, including the USA, Canada, and even New Zealand. In Slovenia, *A. m. carnica* is protected, with measures to conserve the subspecies’ autochthonous domestic population in place. Such efforts depend heavily upon genomic and phylogenetic information. In this study, we sequenced and annotated the mitochondrial genome of a specimen from Slovenia and compared the obtained data with a previously published sample of the *A. m. carnica* from Austria and the closely related Italian honeybee *A. m. ligustica*. We found several features unique to the new mitochondrial genome. We also phylogenetically analyzed the relationship between our sequence and the selected available *A. mellifera* mitochondrial sequences. The acquired position of the sequenced *A. m. carnica* from Slovenia on the phylogenetic tree brings new evidence for close relationships among C and O lineages and reflects their recent historical matrilinear ancestry.

**Abstract:**

The complete mitochondrial genome of the Carniolan honeybee (*Apis mellifera carnica*) from Slovenia, a homeland of this subspecies, was acquired in two contigs from WGS data and annotated. The newly obtained mitochondrial genome is a circular closed loop of 16,447 bp. It comprises 37 genes (13 protein coding genes, 22 tRNA genes, and 2 rRNA genes) and an AT-rich control region. The order of the tRNA genes resembles the order characteristic of *A. mellifera*. The mitogenomic sequence of *A. m. carnica* from Slovenia contains 44 uniquely coded sites in comparison to the closely related subspecies *A. m. ligustica* and to *A. m. carnica* from Austria. Furthermore, 24 differences were recognised in comparison between *A. m. carnica* and *A. m. ligustica* subspecies. Among them, there are three SNPs that affect translation in the *nd2*, *nd4*, and *cox2* genes, respectively. The phylogenetic placement of *A. m. carnica* from Slovenia within C lineage deviates from the expected position and changes the perspective on relationship between C and O lineages. The results of this study represent a valuable addition to the information available in the phylogenomic studies of *A. mellifera*—a pollinator species of worldwide importance. Such genomic information is essential for this local subspecies’ conservation and preservation as well as its breeding and selection.

## 1. Introduction

The western honeybee, *Apis mellifera* L., is a globally distributed, highly polytypic species of great ecological, economic, and agricultural importance. Currently, more than 30 known subspecies are described [1,2,3,4], and their ecosystem services are valued in billions of euros yearly [5]. The world population of *Apis mellifera* is traditionally grouped into four different evolutionary lineages: M, C, O, and A [1,6,7,8]. In addition, Y and S lineages were proposed for the subspecies *A. m. jemenitica* and *A. m. syriaca*, respectively [8,9]. Recently, distinct lineage classification was confirmed also for *A. m. lamarcki* (L lineage—previously grouped under O lineage) and for *A. m. unicolor* (U lineage—previously reported as a member of A lineage), based on the genomic data [10].

Initial morphometric and mitochondrial analyses showed that the C lineage of the North Mediterranean regionconsists of six subspecies: *Apis mellifera adami* (found on Crete)*, A.m.* *carnica* (considered native to Slovenia, Croatia, Bosnia and Hercegovina, Serbia, Montenegro, Southern Austria, and parts of Hungary, Romania, and Bulgaria, but also distributed globally as a consequence of human activity), *A. m. cecropia* (native to Greece), *A. m. cypria* (native to Cyprus), *A. m.* *ligustica* (originally native to the Italian peninsula, but also distributed across the globe), and *A. m. macedonica* (distributed in Bulgaria, Greece, North Macedonia, and Ukraine) [1,11,12]. Recent findings suggest that other subspecies belong to the C lineage, including *A. m. caucasica* and *A. m. carpatica* [13], but their statuses may not be concordant when comparing morphometric and molecular analyses. 

Short segments of mitochondrial DNA have been widely used as DNA markers for decades. Several specific mitochondrial regions became favoured for their particular usefulness in species discrimination, phylogeographic, and evolutionary studies [14,15,16]. In honeybees, the intergenic region located between protein coding genes *cox1* and *cox2*, named *tRNA^Leu^–cox2*, is widely used to distinguish honeybee lineages based on genomic sequence variation [17] and through the AFLP technique, known as the *Dra*I test [18], in which the amplified PCR fragment is digested by the *Dra*I restriction enzyme, resulting in specific restriction profiles. A recent comprehensive study of haplotype occurrence on the mitochondrial genome level between lineages A, M, and C of *A. mellifera* has shown that differential mutation rates in different partitions of the mitogenome may produce incongruent results [19]. Thus, *tRNA^Leu^–cox2* as a marker may not provide easy-to-interpret information for phylogeny inference in honeybee colonies and using complete mitochondrial sequences as a more comprehensive dataset may be beneficial.

In comparison to the nuclear DNA, mitochondrial DNA is predominantly linked and inherited via maternal lineage, which is also true for honeybees [20]. This allows monitoring of the maternal pedigree, and it is useful as a genetic marker in evolutionary and population biology [14,21]. Additionally, it lacks genetic recombination and has a high evolutionary rate (reviewed in [22]). The sequence of the first complete honeybee mitochondrial genome was published in 1993 and was based on the Italian honeybee, *A. m. ligustica* [23]. Since then, the sequencing technologies and bioinformatic software have been developing rapidly, allowing the mitochondrial genome sequencing and annotation to bloom. Complete mitochondrial genomes of more than half of the recognised subspecies of *Apis mellifera* have been published to date [24,25,26,27,28,29,30,31,32,33,34,35,36,37,38,39,40,41,42,43,44], and several studies have provided insight into the phylogenetic relationships, using complete mitochondrial genome sequences [45,46]. Complete mitochondrial genomes of honeybee subspecies were also used in an effort to resolve the controversies surrounding the geographical origin of the Western honeybee [9].

The Carniolan honeybee (*Apis mellifera carnica*) is an indigenous subspecies in South-Central Europe and arguably the second most popular subspecies in beekeeping worldwide [47]. Beekeepers are keen to keep this subspecies due to its favourable phenotypic traits such as its gentle behaviour, high honey yield, and the generous spring building of the colony [1]. Its native distribution has been defined by the last glacial maximum, more precisely from the Adriatic coast and Prokletije in the South, the Alps in the West, and the Carpathian Mountains in the North [1]. At the margins of its natural range there are the zones of hybridization with other subspecies: *A. m. ligustica* [48] in the West, *A. m. mellifera* [49] in the areas adjacent to the Carpathian mountain range, and *A. m. macedonica* [50] in the south of the Balkan Peninsula. The Carniolan honeybee is named after the Duchy of Carniola, the former province of the Austro-Hungarian Empire, within which the *locus classicus* of the taxon is located and which is now within the borders of Republic of Slovenia [51], making the latter the country of origin of *A. m. carnica*. The importance of the Carniolan honeybee has more recently been highlighted by the Treaty of Accession of Slovenia to the EU, which included an important provision in one of its annexes: basis for legal protection of the autochthonous domestic population of »Carnica«. This is projected into various laws and bylaws governing breeding programs and animal trafficking in Slovenia, thus preventing inbound human mediated trade and input of the colonies with alien pedigree [52].

Here, we present the sequenced and annotated complete mitochondrial genome of *A. m*. *carnica* from Slovenia, hereafter referred to as “SICarnica”. To our knowledge, this is the first published, complete mitochondrial sequence of this subspecies from its *locus classicus*. We provide a description and the basic characteristics of the complete mitochondrial genome, and we make comparisons with the reference mitochondrial genome from the closely related *A. m. ligustica,* hereafter referred to as “REFLigustica”. Another mitochondrial genome of *A. m. carnica* is available from an Austrian specimen (hereafter referred to as “ATCarnica”), which was obtained from the Ruttner Bee Collection at the Bee Research Institute in Oberursel, Germany [37]. We depicted the differences in mitochondrial genomes between these two specimens as well. We also performed phylogenetic analyses to present the position of our *A. m. carnica* among closely related specimens. We reveal the phylogenetic relationships among samples from the C and O lineages, based on complete mitogenomic sequences, and discuss the reasons for the deviation from the previously accepted view.

## 2. Materials and Methods

### 2.1. Sample Collection

Authentic Carniolan honeybee material was obtained from the colony of a Slovenian beekeeper and a queen bee breeder (Semič, SE Slovenia) within the framework of a government-sponsored breeding program for the Carniolan honeybee. All colonies in that apiary matched the standards set up by the breeding program for the *A. m. carnica* [53]. Samples were collected in July 2019 from a colony headed by a queen with pedigree number 842-2017 and marked with yellow opalith plate number 51. This particular queen was descended on the maternal side from Dame-of-Queens, pedigree number 549-2015, and from Sire-of-Queens, pedigree number 26-2013, on the paternal side. The queen was mated at the Rog-Ponikve mating station (Bela Krajina, Slovenia; »Sire-of-queens«; pedigree number 460-2014) in 2017. The origin of this particular line goes back to 2007, according to the Original Herdbook. 

We sampled live drones, workers, and drone pupae. These samples were either immediately snap-frozen in liquid nitrogen or stored in absolute ethanol and stored at −140 °C. From ethanol-stored samples, either the flying muscles of mature drones or de-shelled pupae were obtained for DNA extraction procedures.

### 2.2. DNA Extraction

DNA extraction was performed at the National Genomics Infrastructure (NGI) hosted by SciLifeLab. Snap-frozen pupae were thawed and homogenized in a nuclei isolation buffer (10 mM Tris, pH 9.4, 60 mM NaCl, 10 mM EDTA, pH 8, 0.15 mM spermine tetrahydrochloride, 0.15 mM spermidine trihydrochloride, 0.5% Triton X-100, 0.1% 2-mercaptoethanol) using a 1.5 mL disposable homogenizer. Isolated nuclei were resuspended in a CTAB lysis buffer (100 mM Tris, 20 mM EDTA, 2% CTAB, 1.4 M NaCl, 1% PVP10, 1% PVP40, 2% 2-mercaptoethanol, and 1.13 µg/µL Puregene Proteinase K) and incubated on a Thermomixer ON with intermittent mixing (300 rpm, 10 s/10 min, 65 °C) followed by RNase treatment (1 h, 37 °C). DNA was extracted using a standard phenol:chloroform:isoamyl alcohol protocol, and the pellet was resuspended in 100 µL of TE buffer. DNA concentration was measured using the Qubit dsDNA BR Assay Kit, and purity was assessed by UV measurements using the Trinean Dropsense system. Based on quantity and quality of isolated DNA, one drone pupa sample was selected for whole genome sequencing (WGS).

### 2.3. Whole Genome Sequencing and Assembly

Nanopore WGS was performed at the NGI. The sequencing library was prepared using the LSK-109 ligation kit, and sequencing was performed on the PromethION platform using the R9.4 flow cell. Basecalling was done using the Guppy v.3.2.10 (Oxford Nanopore Technologies, Oxford, UK). Fragments smaller than 25 kb were eliminated using the Circulomics Short Read Eliminator kit, and the remaining sample was sheared to 75 kb using the Megaruptor 3 system (Diagenode), prior to library prep. For quality control after the completion of sequencing, the raw signal intensity data were basecalled using Guppy v3.6.0. Only reads with a mean qscore (quality) greater than 10 and a read length greater than 25 kb were used for assembly.

For genome polishing purposes, the DNA from the same sample was analyzed using Illumina sequencing of linked-reads obtained by the 10× Genomics Chromium platform. Briefly, sequencing libraries were prepared from 0.6 ng of DNA using the Chromium Genome Reagent Kit v2 (cat# 120257/58/61/62, 10× Genomics), according to the manufacturers’ protocol (#CG00043 Chromium Genome Reagent Kit v2 User Guide, 10× Genomics). Sequencing was performed on a NovaSeq SP flowcell, paired-end 150 bp read length, using v1 chemistry on a NovaSeq 6000 system (Illumina).

Flye [54] was used to assemble the Nanopore reads under default parameters except “–iterations 2” and “–min-overlap 2000”. The initial assembly was polished using the raw nanopore reads with a mean qscore > 10 and a read length > 1 kb with Racon version 1.4.11 [55], and for one round with Medaka [56] from Oxford Nanopore Technologies. Afterwards, reads from 10× Genomics Chromium Illumina sequencing were aligned to the assembly using Long Ranger version 2.1.0 (10× Genomics). The alignment files were then used by Pilon version 1.22 [57] for polishing. The presence of potential misassemblies was investigated using Tigmint version 1.1.2 [58], and none were detected. Completeness of the genome assembly was evaluated using Benchmarking Universal Single-Copy Orthologs (BUSCO) version 4.0.5 [59] with the Hymenoptera odb10 lineage gene set.

### 2.4. Complete Mitochondrial Genome Retrieval and Annotation

The complete mitogenome of SICarnica was retrieved from the resulting contigs using BLAST search against *Apis mellifera* mitochondrial sequence. Two identified contigs (9.634 and 6.832 bp long) were circularised using Geneious Prime 2021.0.3 [60].

The assembled mitogenome was annotated using Liftoff [61], with annotation of *A. m. ligustica*—GenBank accession number NC_001566 (“REFLigustica”) [23]—used as the reference. Additionally, two tRNA annotation entries (tRNA-Ser and tRNA-Gln), which were not transferred by Liftoff, were added manually after the BLAST search [62] of their respective REFLigustica sequences against the new SICarnica mitogenome.

All protein coding genes (PCGs) were translated into amino acids (invertebrate mitochondrial code) and checked for stop codons. All overlaps of annotated regions were manually curated and corrected if necessary. Secondary structures of annotated tRNAs were predicted using ARWEN [63] and subsequently manually inspected for conformity with known honeybee tRNA structures.

To assign newly obtained mitogenome to an evolutionary lineage, *tRNA^Leu^–cox2* mitotype sequence was extracted and the BLAST search against NCBI nucleotide collection (nt) database was performed. »Filtering for low-complexity regions« and »mask for lookup table only« were switched on in the algorithm parameters of the search. The most significant match with the sequence query was selected for mitotype determination. 

### 2.5. Comparisons between Mitochondrial Genomes

We compared the obtained mitogenome (SICarnica) with the mitogenomes of two closely related and morphologically reliable specimens, both available in GenBank: *A. m. ligustica* (“REFLigustica”—GenBank repository number NC001566) which is a reference mitogenome of the western honeybee and part of BioProject PRJNA13343, and *A. m. carnica* mitogenome (“ATCarnica”—GenBank accession number MN250878), which belongs to *A. m. carnica* worker honeybees obtained from the Ruttner Bee Collection at the Bee Research Institute in Oberursel, Germany (Voucher No. 1668, Dr. M. Meixner, 1990, Austria, 46°37 N, 14°19 E) [36]. The Ruttner Bee Collection contains morphometrically determined specimens and is a referential source of *A. mellifera* subspecies.

To determine the differences between the mitogenomes we made a pairwise alignment of the sequences using the MAFFT plug-in [64] in Geneious. We then determined the differences between the aligned sequences using Geneious. Both the alignments and the variant calling output were visually inspected. Alignment was also repeated using ClustalOmega [65] and compared with the MAFFT output to check for consistency between the alignments.

### 2.6. Phylogenetic Analyses

We used two different datasets to infer phylogenetic analyses. The first phylogenetic dataset comprised a selection of 29 complete mitochondrial genomes of *Apis mellifera*. The complete mitochondrial genome sequences were obtained from GenBank (NCBI) on 1 April 2021. This dataset includes all currently available *A. mellifera* subspecies and additional samples belonging to the C lineage relevant to our study and are based on three criteria. The selection criteria for inclusion of these mitogenomes into the analyses were: (1) the inclusion of all the available subspecies, (2) the subspecies was sampled in the geographic area of its known origin, and (3) the sample was taken from the Ruttner’s collection, when available. We also included some “strains” such as “buckfast” and “Italian” instead of a subspecies; in this case, we kept the authors’ own designations. 

The complete mitochondrial genome of the closest known species Asian honeybee *Apis cerana* ([66]; Genbank accession number: GQ162109) served to root the phylogenetic trees. A detailed list along with the references is available in Supplementary Table S1.

Complete mitochondrial genomes were aligned using an auto-selected algorithm in the MAFFT plug-in in Geneious. Phylogenetic analyses were inferred using both aligned complete mitogenomic sequences as well as aligned partial mitogenomic sequences. Three different approaches were then used to obtain partial datasets: (1) arbitrary inclusion of 13 PCGs only, (2) using the GBlocks server (Institut de Biologia Evolutiva, Spain), with the least stringent conditions applied to exclude poorly aligned regions and most divergent positions of the alignment [67], and (3) using the software Noisy to exclude phylogenetically uninformative homoplastic sites [68]. In the next step, phylogenetic analyses were inferred on complete sequences: (1) the maximum likelihood approach by RAxML and (2) the Bayesian inference (BI) approach by Phylobayes or MrBayes. For partial datasets, MrBayes was used to infer phylogenetic relations. All the analyses were performed at CIPRES Science Gateway v3.3 [69].

A Maximum likelihood (ML) analysis was performed using RAxML-NG v 0.9.0 [70] and was run with automatic MRE bootstopping set to a maximum of 3000 bootstraps, with a 0.03 cutoff and a GTR + F0 + I + G4m model. Bootstrapping converged after 550 replicates. The maximum likelihood tree with bootstrap support values on the nodes was calculated. 

Using the PhyloBayes-MPI v1.7b software [71], Bayesian phylogenetic analysis under the site-heterogeneous mixture model CAT-GTR with a gamma distribution of rates across sites was also performed. Constant columns were removed from the alignment (-dc option) and the maxdiff acceptable was set to under 0.1. Two independent MCMC chains were run, and the convergence was checked in Tracer v1.7.1 [72]. A majority rule consensus tree with posterior probabilities on the nodes was calculated.

Bayesian analysis using MrBayes v3.2.7a [73] was performed in parallel using two MCMCMC algorithms. Each algorithm had three warm chains and one cold chain. The best substitution model was assigned using the jModelTest2 v.2.1.6 under cAIC [74,75] and was set to GTR + I + G in all of the analyses. The number of generations was adjusted to 2,000,000. We sampled every 1000th generation, and the first 25% of the sampled trees were discarded (burn-in). We calculated a 50% majority rule consensus tree from the remaining trees.

Evolutionary distances were estimated using the Jukes–Cantor model [76] implemented in MEGAX software [77]. The rate variation among sites was modelled with a gamma distribution (shape parameter = 1), and ambiguous positions were removed for each sequence pair (pairwise deletion option). 

A distance method called the Automated Barcode Gap Discovery (ABGD) [78] was used for the delimitation of intraspecific groups. Aligned sequences were analysed using default settings under either the Jukes–Cantor [76] or the K80 Kimura [79] measure of distance.

Our study focusses on reliably identified specimens. To highlight issues regarding the identification of subspecies and their designation into lineages, we performed an extended phylogenetic analysis based on the available complete mitochondrial genomes that were available from GenBank (NCBI) on 29 March 2021. During the selection of the mitochondrial sequences, we made two exceptions: (1) we omitted the mitochondrial genomes of African *A. m. mellifera,* for which we were unable to extract the reliable subspecies assignment, and (2) from the obtained mitochondrial sequences we retained only unique sequences and discarded all duplicates from the further analysis. Using these criteria, 172 mitochondrial genomes were aligned for the extended phylogenetic analysis using an auto-selected algorithm in the MAFFT plug-in in Geneious. Since the control region was absent from a set of mitochondrial sequences (GenBank accession numbers OM203219 to OM203348), we excluded this fragment from the mitochondrial genome alignment. Phylogenetic analysis was then inferred using MrBayes v3.2.7a, as described above. The number of generations was set to 3,000,000. The ABGD method for intraspecific group delimitation was applied to the extended dataset as described above. The phylogenetic tree and the results from ABGD are presented in Figure S7.

## 3. Results and Discussion

### 3.1. Main Characteristics of the New Mitochondrial Genome of Apis m. carnica from Slovenia

The mitochondrial genome (SICarnica) was obtained in two contigs only, which greatly assisted reassembly. The genome is a circular closed loop 16,447 bp long. The proportions of A, T, G, and C are as follows: A (7114—43.3%), C (1561—9.5%), G (905—5.5%), and T (6867—41.8%). GC content is 15.0%. Strong AT bias (85%) is also characteristic for many other mitogenomes of the same and closely related subspecies [25,27,31,45,80]. 

**Figure 1:**
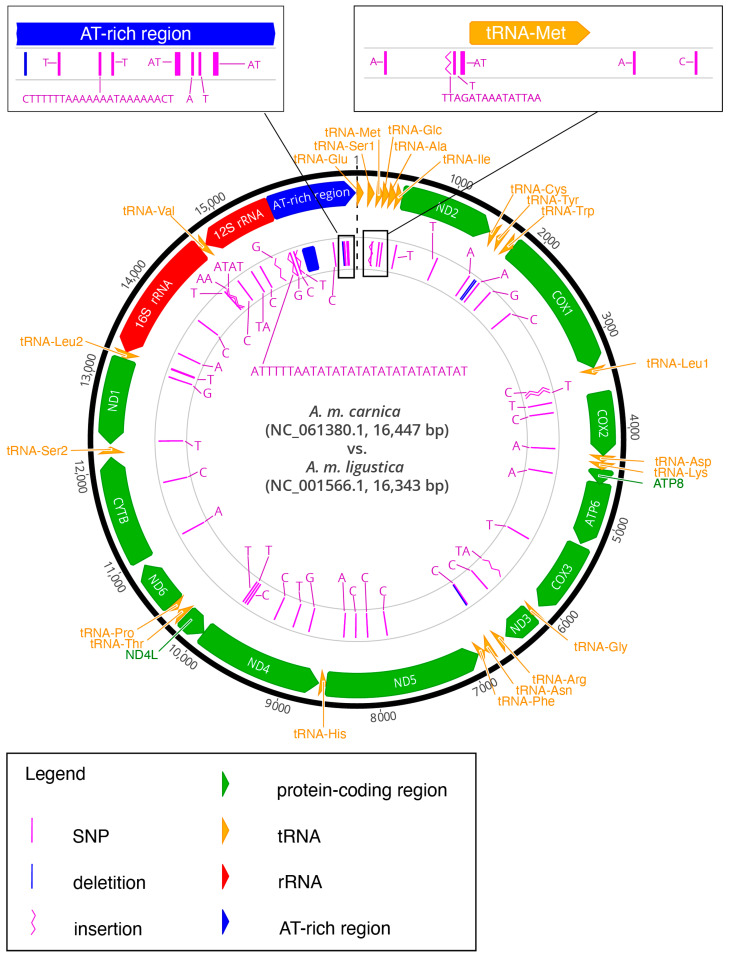
The circular representation of the complete mitochondrial genome of *A. m. carnica* from Slovenia (SICarnica) and its comparison to the mitochondrial genome of *A. m. ligustica* (REFLigustica). The newly obtained mitochondrial genome of SICarnica is presented by the outer circle with all corresponding annotations. Either light- or heavy-strand coding is indicated by the direction of the arrows. The transparent inner circle presents the mitochondrial genome of REFLigustica. The detected differences in comparison to the mitochondrial genome of *A. m. carnica* are outlined. The two most variable regions (the AT-rich region and the region around tRNA-Met) are enlarged to enable a detailed view of the comparisons.

Figure 1 represents the mitogenomic sequence annotation characteristics and comparison with the mitogenome of *A. m. ligustica* (REFLigustica; NC_001566). The newly obtained mitochondrial genome of SICarnica is presented by the outer circle with all corresponding annotations. Either light- or heavy-strand coding is indicated by the direction of the arrows. The transparent inner circle represents the mitochondrial genome of REFLigustica, the most closely related subspecies, and the detected differences in comparison to the mitochondrial genome of *A. m. carnica* are outlined. The two most variable regions (the AT-rich region and the region around tRNA-Met) are enlarged to enable a detailed view of the comparisons.

The mitochondrial genome comprises 37 genes, structurally resembling other related species [22]. Of the 13 PCGs, 9 are encoded on the light strand (*nd2, cox1, cox2, atp8, atp6, cox3, nd3, nd6,* and *cytb*), and 4 are encoded on the heavy strand (*nd5*, *nd4*, *nd4l*, and *nd1*). Genes *atp8* and *atp6* overlap by 19 bases. Different start codons are used; one gene starts with ATC, three with ATG, three with ATA, and six with ATT. All but one PCG end with the TAA stop codon. The only exception is *cox1*, which ends with a truncated stop codon T (Table S2). The 22 tRNAs vary in length from 63 bp (tRNA-Ser, tRNA-Gln) to 78 bp (tRNA-Thr). Fourteen tRNAs are encoded on the light strand (tRNA-Glu, tRNA-Ser1, tRNA-Met, tRNA-Glc, tRNA-Ala, tRNA-Ile, tRNA-Trp, tRNA-Leu1, tRNA-Asp, tRNA-Lys, tRNA-Gly, tRNA-Asn, tRNA-Thr, and tRNA-Ser2), and 8 are encoded on the heavy strand (tRNA-Cys, tRNA-Tyr, tRNA-Arg, tRNA-Phe, tRNA-His, tRNA-Pro, tRNA-Leu2, and tRNA-Val). Ribosomal genes (16S rRNA and 12S rRNA) are both encoded on the heavy strand. The 16S rRNA gene is 1367 bp long (40.5% A, 44.0% T), whereas the 12S rRNA is 784 bp long (42.0% A, 39.7% T). The AT-rich control region (50.8% A, 46.1% T) encompasses a 945 bp stretch of the mitogenome.

After the initial annotation of the SICarnica mitogenome, the *cox1* gene overlapped with tRNA-Leu coding sequence by five bases. Comparison with the ATCarnica *cox1* sequence and the corresponding annotation showed that the overlap was caused by a truncated stop codon (T instead of TAA) in the *cox1* sequence, followed by an apparent full stop codon (TAA) within the tRNA-Leu sequence. The annotation was corrected to match that of ATCarnica, effectively shortening the *cox1* sequence by five base pairs and eliminating the overlap. A complete and annotated nucleotide sequence was deposited in GenBank (https://www.ncbi.nlm.nih.gov/genbank/) (accessed on 1 April 2022) under the accession number MW811175 and included into NCBI Reference Sequence database (RefSeq) under the accession number NC_061380.1 (https://www.ncbi.nlm.nih.gov/refseq/) (accessed on 1 April 2022).

As is the case in other *A. mellifera* mitogenomes, the structure of the SICarnica mitogenome differs from the proposed ancestral insect mitogenome [22]. The ancestral ordering of tRNA coding genes immediately follows the control region: tRNA-Ile, tRNA-Gln, and tRNA-Met is changed in SICarnica mitogenome to: tRNA-Glu, tRNA-Ser, tRNA-Met, tRNA-Gln, tRNA-Ala, and tRNA-Ile. Moving away from the control region, between *nd2* and *cox1*, the ancestral ordering of tRNA-Trp, tRNA-Cys, and tRNA-Tyr is replaced by tRNA-Cys, tRNA-Tyr, and tRNA-Trp. Separating *cox2* and *atp8* are tRNA-Asp and tRNA-Lys, instead of tRNA-Lys and tRNA-Asp. tRNA-Ala, tRNA-Arg, tRNA-Asn, tRNA-Ser, tRNA-Glu, and tRNA-Phe occupy the area flanked by *nd3* and *nd5* in the ancestral insect genome. In their stead in SICarnica mitogenome, there are tRNA-Arg, tRNA-Asn, and tRNA-Phe. The protein coding gene order corresponds to the proposed ancestral insect mitogenome [22]. 

Based on the characteristics of the *tRNA^Leu^–cox2* region, the SICarnica mitogenome belongs to C2e mitotype (the most significant BLAST hit with the sequence query was JQ977702). This mitotype is common in the *A. m. carnica* population in Slovenia as well as present throughout its distribution range [81,82]. 

### 3.2. Comparison to Mitogenomes from Closely Related Specimens

The content and arrangement of genes in the mitogenome of SICarnica are similar to the content and arrangements found in the mitogenomes of REFLigustica and ATCarnica. We performed three different comparisons between the mitochondrial sequences of SICarnica, ATCarnica, and REFLigustica to detect variable sites by: (1) the pairwise alignment of SICarnica and REFLigustica (Figure 1 and Table S3), (2) the pairwise alignment of SICarnica and ATCarnica (Table S4), and (3) the multiple sequence alignment of SICarnica, ATCarnica, and REFLigustica (Table 1, Table 2 and Table S5). In total, there are 64 variable sites between REFLigustica and SICarnica (Figure 1 and Table S3). The comparison with the REFLigustica mitogenome shows differences at 4 sites in the tRNA genes, 24 in the PCGs, 11 in the rRNA coding genes, 14 in the control region, and 11 in the intergenic regions. The differences between the sequence of the SICarnica mitogenome and that of ATCarnica amount to 59 variable sites. A total of 6 are in the tRNA genes, 20 in the PCGs, 7 in the rRNA genes, 18 in the control region, and 8 in the intergenic regions (Table S4). 

The control region is the most variable part of the mitogenome. Of note are the different control region lengths: 945 bp (SICarnica) versus 832 bp (ATCarnica) and 827 bp (REFLigustica). This variability is mostly due to different stretches of As and Ts, tandem repeats of the AT motif, and several insertions or deletions. In mammals and several other vertebrates, as well as in some invertebrate taxa, the control region has proved useful in phylogenetic inferences [83,84,85]. However, in insects, the utilization of this region is problematic [86,87]. A recent study of the structure and genetic variation of the control region in different populations of *Apis mellifera* revealed that it contains limited phylogenetic signals and may not be suitable to resolve relationships within the population [88]. 

Besides length variation in the control region, several notable differences in length are also observed in the rRNA genes. The 16S rRNA, which is 1371 bp long in ATCarnica and REFLigustica, is 4 bp shorter in SICarnica (1367 bp). The 12S rRNA is also shorter in SICarnica, having a length of 784 bp, compared to 785 bp in ATCarnica and 786 bp in REFLigustica. 

In the comparison of all three mitogenomes, we were able to characterise unique differences of SICarnica in relation to ATCarnica and REFLigustica, as well as the characteristics that differentiate the two closely related specimens of *A. m. carnica* (SICarnica and ATCarnica) from *A. m. ligustica* (REFLigustica). Selected data are presented in Table 1 and Table 2; complete data are presented in Tables S5 and S6. There are 44 differences unique to SICarnica in comparison to both ATCarnica and REFLigustica. Most are positioned in the control region, rRNA and tRNA genes, and in PGCs, while eight unique differences are present in the intergenic regions (Table 1 and Table S5). Notably, all but one of the changes at these positions in DNA sequences are shared by ATCarnica and REFLigustica, the one exception being a deletion/insertion located in the 16S rRNA gene at the aligned position of 14,589 bp (Table S5). While it is tempting to suggest that differences described above represent unique characteristics of the mitochondrial genome of *A. m. carnica* from Slovenia, the extensive sampling throughout the natural distribution range of this subspecies is required to make such conclusions.

Table 1 presents the selection of changes within PCGs unique to SICarnica in comparison to both ATCarnica and REFLigustica that result in eight substitutions in amino acid residues predicted in SICarnica proteins. The product of *nd4* is most significantly affected, exhibiting two amino acid substitutions in comparison to ATCarnica and REFLigustica (Table 1). To further explore these alterations, specific oligonucleotide primer pairs could be constructed to amplify these regions and thus pinpoint the nucleotide changes on a greater number of samples. Taken together, our findings promote and call for further inquiry into the mitochondrial diversity of distinct *A. m. carnica* populations throughout natural range of occurrence. 

Twenty-three genetic features that are common to both *A. m. carnica* mitogenomes but differ from the REFLigustica mitogenome are presented in Table 2 and Table S6. These changes might be the reference features for delimiting these two closely related subspecies in the C lineage. Furthermore, three substitutions in amino acid residues are predicted in comparisons between *A. m. carnica* and *A. m. ligustica* subspecies. Amino acid changes in mitochondrial proteins may affect energy metabolism and could have consequences for the fitness of a honeybee colony. These missense mutations may be considered important as signatures of natural selection and reflective of an adaptation to local environment conditions [89]. A more detailed study on a larger number of specimens of both subspecies is needed to confirm whether the predicted amino acid changes are characteristic of each subspecies and influence the metabolism, or are merely a result of the variability within and among populations.

The evolutionary distance between the mitogenomes of SICarnica and ATCarnica and between SICarnica and REFLigustica was similar (0.00247 and 0.00278, respectively). In comparison, the evolutionary distance between the mitogenome of SICarnica and all the other *A. mellifera* mitogenomes was 0.01067.

### 3.3. Phylogenetic Relationships between and within the Evolutionary Lineages

First, to validate the newly obtained SICarnica mitogenome and to identify its phylogenetic position, we used the 13 PCGs dataset for the phylogenetic analysis. Selection of exclusively protein-coding genes is a common practice in mitochondrial phylogenetics [22] and an approach that is also used in some studies of *A. mellifera* [9]. The BI analysis showed a sister relationship between C and O lineages (Figure S4). Such a result is in agreement with the previously supported view [37] and verifies the correctness of the mitogenomic information.

**Figure 2:**
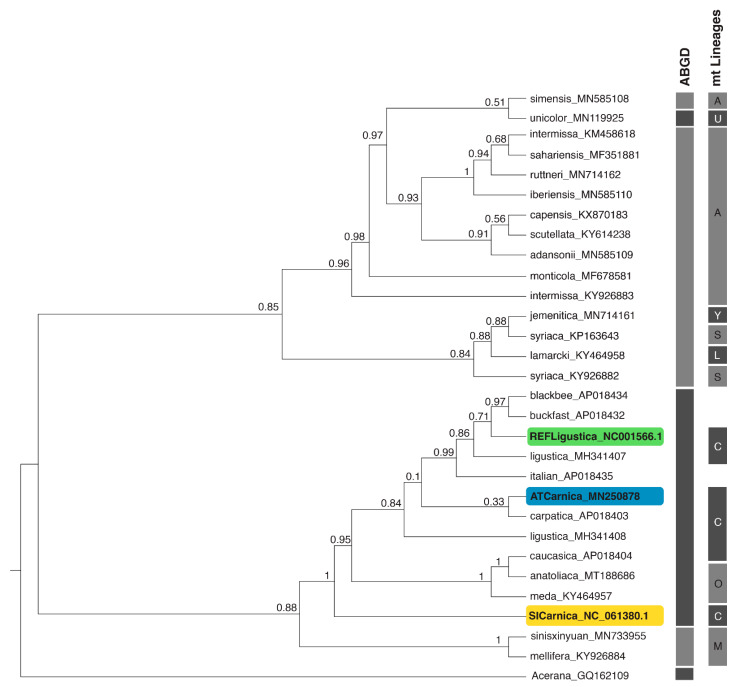
Maximum likelihood phylogenetic analysis of the selected *Apis mellifera* subspecies based on complete mitogenome sequences. Branch lengths are omitted. Bootstrap support values are presented on the nodes. Colored marks emphasize the phylogenetic position of the complete mitochondrial genome of *A. m*. *carnica* from Slovenia (SICarnica) in relation to REFLigustica and ATCarnica. Vertical bars at the right edge indicate group delimitation using the ABGD method. Each subspecies belonging to evolutionary lineage is represented by the right-most vertical bars and the letters M, C, O, S, L, Y, A and U (marked as mt Lineage). Lineage was assigned according to the literature, when provided. Names of the samples include the subspecies or strain name and the GenBank accession number.

Then, based on complete mitogenomic sequences (Table S1), we performed ML (Figure 2 and Figure S1) and BI (S2, S3) phylogenetic analyses. The length of the alignment of complete mitogenomic sequences was 17456 bp. The resulting tree topologies introduced different perspectives on the phylogenetic relationships of *A. mellifera* subspecies, departing from the traditional view [9,24]. These analyses positioned SICarnica on the outermost branch of a clade containing both C and O lineage representatives, making this clade paraphyletic due to O lineage being nested within C lineage. In lieu of these results, additional phylogenetic analyses were carried out with partial sequence datasets to check the effect of character exclusion/inclusion on the tree topology. While the dataset of 13 PCGs only selection was arbitrary (11111 bp, Figure S4), two objective character exclusion approaches were also used (GBlocks server algorithm, 16286 bp, Figure S5; Noisy software, 13867 bp, Figure S6). All the phylogenetic analyses confirmed the position of the newly sequenced SICarnica from Slovenia within the C lineage of the *A. mellifera* subspecies clade (Figure 2 and Figures S1–S6). While partial dataset using PCGs only yielded sister position between C and O lineages, all other phylogenies produced a paraphyly of C and O lineages, disregarding the character exclusion selection approach or phylogenetic principle used. Both algorithmic exclusions of the characters (GBlocks or Noisy) yielded similar BI topologies to those produced by using complete mitogenomic sequences and are less like the analysis produced by PCGs only dataset. In both cases the less informative sites are removed but the principle of exclusion differs. While GBlocks removes poorly aligned and most divergent sites [67], Noisy removes randomised sites from a pre-computed alignment [68].

The distance-based delimitation method ABGD [78] for delimitation of intraspecific groups using either the Jukes–Cantor or the K80 Kimura measure of distance supported the clades obtained with BI and ML analyses. Using a prior maximal distance P = 1.00 × 10^−3^, six groups were recognised regardless of the dataset used (complete mitogenomic sequences or partial datasets). The groups as recognised by ABGD analysis are represented by the right-most vertical bars in Figure 2. From the bottom up, the outgroup species *A. cerana* represents one group. The second group consists of M lineage representatives. The third group contains both O and C lineage subspecies. The fourth group consists of all the representatives of the A, L, and Y lineages, except for the two African subspecies *A. m. simensis* (A lineage) and *A. m. unicolor* (U lineage). These two subspecies represent one separate group each. Despite the fact that the resulting topology from the PCGs only dataset is more in congruence with the traditional perception of relationships among *A. mellifera* lineages and subspecies, the ABGD analysis shows that genetic distances between C and O lineages using PCGs only dataset also does not qualify to form distinct intraspecific groups. Furthermore, such subjective exclusion of large parts of the mitochondrial genome from the phylogenetic analysis is difficult to justify. It had been shown that the inclusion of tRNA and rRNA genes into phylogenomic analyses improves nodal confidence and stabilizes highly variable backbone relationships [22]. It may be sensible to make a comprehensive re-evaluation of the evidence from both the morphology and genetics of C and O lineage representatives to better define their relationships. The information from complete mitochondrial DNA in honeybees is believed to be predominantly of maternal origin, and does not include any paternal information. Thus the upgrade of phylogeny using the nuclear DNA would be beneficial.

Figure 2 represents the ML tree inferred from complete mitogenomic sequences by RAxML-NG analysis with bootstrap support on the nodes. Since the branch lengths in the *A. mellifera* group are extremely short, we omitted branch lengths and used proportionally transformed branches to ensure a clear visual presentation of the tree topology. Trees with computed branch lengths and topologies recovered by ML and BI analyses are presented as a supplement (Figures S1–S3). Some of the BI analyses produced uncertain relationships with low posterior probability values at the base of the trees but major clades were always recovered (Figure 2 and Figures S1–S6).

All *A. mellifera* samples form two distinct clades with sister relationships in the ML phylogenetic tree. In the first clade, representatives of A, Y, L, and U evolutionary lineages are placed. The second clade contains samples belonging to M, O, and C lineages. Clear separation of M, O, and C lineages from A, L, and U lineages was also confirmed in a recent comprehensive genomic study [10], suggesting the concordant phylogeny of lineages is supported by both nuclear and mitochondrial data.

The first clade is further divided into two well-supported paraphyletic groups. The first group contains all subspecies of African origin, except for *A. m. lamarcki*, aligning together A and U lineages. Sister relationships of the subspecies *A. m. simensis* from Ethiopia (A lineage) and *A. m. unicolor* (recently proposed as distinct lineage named U) [10] from Madagascar are weakly supported in ML phylogenetic tree, but this relationship remains valid (posterior probability values from 0.79 to 1.0) in all other phylogenetic analyses (Figures S2, S3, S5 and S6), with the exception of using dataset with regions containing PCGs only (Figure S4). The highest posterior probability of this split (1.0) is obtained in BI phylogenetic analysis using dataset with regions excluded by Noisy software (Figure S6). We also included two specimens of *A. m. intermissa*, a highly admixed subspecies [10], native to Tunisia, Algeria and Morocco [1,11,90] in our analyses. These two samples do not cluster together but occupy distant positions within the subclade. The second subclade comprises three subspecies, distributed in North Eastern Africa and the Middle East. *A. m. lamarckii* is a subspecies native to Egypt that was previously reported as the O lineage [13], but due to its unique genetic signatures [10], proposed classification as a distinct lineage (L). *A. m. lamarckii* is consistently clustered together with samples from the S and Y lineages (*A. m. syriaca* and *A. m. jemenitica*), with high support values on the nodes in all of our phylogenetic analyses (Figure 2 and Figures S1–S6) and is phylogenetically distant from other representatives of O lineage. Such a result is in concordance with the results of another recent study on the origin of the Western honeybee, based on mitochondrial genomes [9] where *A. m. lamarckii* was located as a sister taxon to *A. m. syriaca*; however, this relationship was not recovered in all the phylogenies and node support was weak. One possible explanation is the exclusion of the *A. m. jemenitica*, another subspecies with an unclear lineage origin, from their analysis. Previous studies based on mitochondrial haplotypes attributed *A. m. jemenitica* to either O or A lineage [91]. This subspecies forms a close relationship with *A. m. syriaca* and *A. m. lamarckii* in all our analyses with consistent high support on nodes. Similar results are also evident from a few previous studies [32,41]. The study of population structures using 11.8 million SNPs detected admixture within *A. m. syriaca*. The explanation for high levels of hybridisation is the geographic location of *A. m. syriaca*, which is within the contact zone between African lineages A and L, and Middle Eastern lineages Y and O [10]. 

The second clade comprises samples from M, O, and C lineages. The subclade representing the M lineage contains two subspecies, *A. m. mellifera* and *A. m. sinisxinyuan*. A sister relationship between *A. m. mellifera* and *A. m. sinisxinyuan* has been confirmed already both by mitochondrial and nuclear sequence analyses [4,10,43] and may imply that this clade was once more diverse and widespread. Both *A. m. sinisxinyuan* and *A. m. mellifera* may be relics. *A. m. sinisxinyuan* is nowadays limited to a small area in China, in the Xinyuan prefecture [4], whereas *A. m. mellifera* is limited to Western and Northern Europe [13]. Both subspecies have adapted to survive harsh winters and temperate climates. The second subclade contains representatives of C (*A. m. carnica, A. m. ligustica*, and *A. m. carpatica*, and *A. m. caucasica*) and O evolutionary lineages (*A. m. meda* and *A. m. antoliaca*). While previous studies placed the O lineage as a sister to the C lineage [9,24], a result matching ours, when using PCGs only (Figure S4), our analyses using whole mitogenomes consistently nested the O lineage within the C lineage (Figures S2, S3, S5 and S6). The two results seem to be contradicting at first but based on previous evidence, O and C lineages are considered very closely related [1]. *A. m. caucasica*, which was previously thought to belong to the O lineage, has been already confirmed to belong to the C lineage, following an analysis of mitochondrial DNA [13]. Thus, the clustering with closely related subspecies *A. m. meda* and *A. m. anatoliaca* within the clade representing the C lineage is not surprising. Furthermore, the earlier investigations of *A. m. anatoliaca* found only little distinction from the C lineage [1,92,93,94]. Thus, based on maternal origin, it is reasonable to conclude that O lineage is a part of C lineage. 

To highlight the issues with the subspecies and lineages, we tested the robustness of the phylogenetic relationships within and between lineages. We performed the phylogenetic analysis with an extended dataset (Figure S7) that included 172 samples, with an aligned length of 16,365 bp. The resulting phylogenetic tree, which also includes intraspecific grouping according to ABGD analysis, is presented in Supplementary Figure S7. Lineage was assigned according to the literature, when provided. Sign * indicates a sample OM203262 being determined as *A. m. ligustica* but belonging to S instead of C lineage [95]. This example clearly shows the discrepancies between subspecies and lineage designation when using matrilineal information only. The basal relationships between the clades changed, despite the high posterior probability values on the nodes. Nevertheless, the samples we used in other phylogenetic analyses (marked with coloured shades) retained their positions within the previously recognised clades. The addition of multiple samples from C and O lineage did not affect the position of the sample of *A. m. carnica* from Slovenia. It is positioned on the outermost branch of a clade containing both C and O lineage representatives, as in other phylogenetic analyses (Figure 2, Figures S1–S3 and S6). The O lineage remains nested within the C lineage. The ABGD analysis recognised eight intraspecific groups which may be delimited. Again, samples belonging to C and O lineages form a single group, indicating that using currently available mitogenomic information, O lineage greatly resembles C lineage.

To further extensively investigate the phylogenetic relationships within the C lineage and between C and O lineages, it is necessary to extend the sampling and gain relevant missing mitogenomic information from other geographically and genetically closely related subspecies, such as *A. m. macedonica*, *A. m. adami*, *A. m. cypria*, and *A. m. cecropia*. Additional genetic information would importantly contribute to our understanding of the local adaptation, distribution, and diversity of honeybees.

## 4. Conclusions

In modern animal husbandry, SNP molecular tools are used to make decisions about a selection plan, depending on the requirements. Often, the offspring performance is predicted according to the identified SNPs of parents, the values of which were calibrated against the parents’ performance. As has been shown in the past, the mitochondrial DNA is also a good starting point for production evaluation, as seen in cattle fertility and beef production [96,97]. However, the reproduction of honeybees has its unique characteristics (reviewed in [98]). The honeybee queen is a single vessel of reproductive material in a colony, and it mates only once during its lifespan with multiple drones that are haploid. The descendant drones carry genotypes of a subsample of the queen’s genetic material. The exception is the mitochondrial genome, which is passed on in its entirety. Drones’ mitochondrial DNA does not get passed on to the offspring, yet the variability of cell respiration chain, which is partially coded by mitochondrial DNA could contribute to drones’ performance during mating attempts [99,100]. It is thus at least hypothetically possible that mitochondrial molecular markers could aid genetic gain in breeding on both paternal and maternal sides.

In this study, we presented a complete mitogenome from the subspecies *Apis mellifera carnica*. This is the first completely covered mitogenomic sequence obtained from a specimen from the native Carniolan honeybee area, where the *Apis mellifera carnica* population is legally protected, and measures are applied to minimise the anthropogenic gene flow of this subspecies from abroad, as well as the presence of hybrids and non-native subspecies [52]. The mitogenomic sequence and annotation was also included into NCBI’s RefSeq collection, which is a comprehensive, integrated, non-redundant, and well-annotated set of reference sequences including genomic, transcript, protein, and RefSeq sequences that form a foundation for medical, functional, and diversity studies [101]. As such, this mitogenomic sequence may serve as a reference mitochondrial genome for this subspecies in future studies. However, additional genomic information is required to confirm the unique sequence features across its population, as indicated in our extended sampling.

The phylogenetic analysis of complete mitochondrial genomes of selected *A. mellifera* subspecies places the newly sequenced mitochondrial genome in an outer branch of a well-supported clade, along with other representatives of the C lineage and, conspicuously, the O lineage (*A. m*. *caucasica*, *A. m. anatoliaca*, and *A. m. meda*) as well, which is nested within the C lineage. The obvious culprit is the use of entire mitogenome information for phylogeny reconstruction instead of the more popular arbitrary selection of using protein coding mitogenomic fragments only. On the other hand, alternative subsetting of the mitogenome dataset with the objective approach using GBlocks or Noisy did support a novel interpretation of the complete mitogenomic data.

The discrepancies [13] appearing between the morphometry and the mitochondrial-DNA-based lineage classifications call for more thorough phylogenetic analyses of the subspecies belonging to the C and O lineages, and our extended analysis suggests this is also needed across other lineages for the *A. mellifera* subspecies. A clear first step would be the complete sequencing of the *A. m. macedonica*, *A. m. cecropia*, and *A. m. cypria* mitochondrial genomes as well as addition of information from nuclear genomes and morphometry. 

The results of this study represent a valuable addition to the information available for phylogenomic studies of the honeybee. They may reflect historical dispersal and isolation, as well as conservation and protection measures for the management of this indigenous honeybee subspecies in Slovenia.

Additionally, the findings are essential for the local subspecies’ conservation and preservation and form the basis for investigations into the molecular mechanisms underlying important phenotypic traits of *A. mellifera*—a pollinator species of worldwide importance.

## Figures and Tables

**Table 1:** Missense mutations representing unique mitogenomic features of *Apis mellifera carnica* from Slovenia (SICarnica) in comparison to the mitogenomes of ATCarnica and REFLigustica.

Aln Position (bp)	Region	SICarnica Position (bp)		ATCarnica Position (bp)		REFLigustica Position (bp)		Amino Acid Change
1113	*nd2*	1095	A	1113	T	1110	T	L → F
1944	*cox1*	1926	A	1936	G	1933	G	N → S
5506	*cox3*	5486	A	5497	T	5495	T	T → S
7849	*nd5*	7821	A	7840	C	7830	C	S → A
9823	*nd4*	9795	C	9814	T	9804	T	D → N
9854	*nd4*	9826	A	9845	T	9835	T	F → L
11,070	*cytb*	11,042	T	11,061	A	11,051	A	I → M
12,344	*nd1*	12,316	C	12,335	T	12,325	T	V → M

**Table 2:** Sites that are constant in both *Apis mellifera carnica* mitogenomes and enable differentiation between *A. m. carnica* and *A. m. ligustica* subspecies. Abbreviations: IR1–IR4: intergenic regions; *nd2*, *atp8*, *nd3*, etc.: PCGs; CR: control region; i.e.: if applicable.

Aln Position (bp)	Region	SICarnica Position (bp)		ATCarnica Position (bp)		REFLigustica Position (bp)		Amino Acid Change (i.a.)
218	IR1	202	A	218	A	217	-	
544	*nd2*	526	C	544	C	541	T	L → F
1592	IR2	1574	G	1592	G	1589	A	
3439	IR3	3420	-	3430	-	3428	C	
4253	*cox2*	4233	G	4244	G	4242	A	V → I
4577	*atp8*	4557	G	4568	G	4566	A	
6376	*nd3*	6348	T	6367	T	6359	C	
6712	IR4	6684	AT	6703	AT	6694	--	
8108	*nd5*	8080	T	8099	T	8089	C	
8894	*nd4*	8866	A	8885	A	8875	G	
9110	*nd4*	9082	A	9101	A	9091	T	F → L
11,796	*cytb*	11,768	T	11,787	T	11,777	C	
13,203	*nd1*	13,175	A	13,194	A	13,184	G	
13,312	16S rRNA	13,284	C	13,303	C	13,293	T	
14,046	16S rRNA	14,018	T	14,035	T	14,025	C	
14,568	16S rRNA	14,540	C	14,557	C	14,547	T	
14,589	16S rRNA	14,559	-	14,578	T	14,568	A	
15,150	12S rRNA	15,115	A	15,139	A	15,129	C	
15,361	12S rRNA	15,325	-	15,349	-	15,340	G	
15,613	CR	15,547	T	15,601	T	15,591	G	
16,204	CR	16,135	T	16,020	T	16,010	C	
16,325	CR	16,255	A	16,141	A	16,129	-	
16,354	CR	16,284	A	16,170	A	16,158	C	

## Data Availability

The sequence and annotation data presented in this study are openly available in GenBank (NCBI) under the accession number MW811175.

## Supplementary Materials

The following supporting information can be downloaded at: https://www.mdpi.com/article/10.3390/insects13050403/s1. Figure S1: Maximum likelihood phylogenetic analysis of selected *A. mellifera* subspecies based on complete mitogenome sequences. Figure S2: Bayesian phylogenetic analysis of selected *A. mellifera* subspecies using PhyloBayes software based on complete mitogenome sequences. Figure S3: Bayesian phylogenetic analysis of selected *A. mellifera* subspecies using MrBayes software based on complete mitogenome sequences. Figure S4: Bayesian phylogenetic analysis of selected *A. mellifera* subspecies using MrBayes software and based on partial mitogenome sequences (PCGs only dataset). Figure S5: Bayesian phylogenetic analysis of selected *A. mellifera* subspecies using MrBayes software and based on partial mitogenome sequences (GBLOCKS dataset). Figure S6: Bayesian phylogenetic analysis of selected *A. mellifera* subspecies using MrBayes software and based on partial mitogenome sequences (Noisy dataset). Figure S7: Bayesian phylogenetic analysis of extended *A. mellifera* subspecies using MrBayes software and based on complete mitogenome sequences excluding control region. Table S1: Samples in phylogenetic analyses. Table S2: PCG start and stop codon usage. Table S3: Pairwise alignment comparison of SICarnica and REFLigustica. Table S4: Pairwise alignment comparison of SICarnica and ATCarnica. Table S5: Differences of SICarnica in comparison to ATCarnica and REFLigustica. Table S6: Differences of *A. m. carnica* (SICarnica and ATCarnica) in comparison to *A. m. ligustica* (REFLigustica).
