# Supplementary material for: The Carniolan Honeybee from Slovenia—A Complete and Annotated Mitochondrial Genome with Comparisons to Closely Related Apis mellifera Subspecies
Source: Insects. 2022 Apr 22;13(5):403. doi: 10.3390/insects13050403 (PMC9146700; doi:10.3390/insects13050403)
Supplement: Supplementary file 1 [file insects-13-00403-s001.zip › insects-1411432-supplementary-Figure S1.pdf]

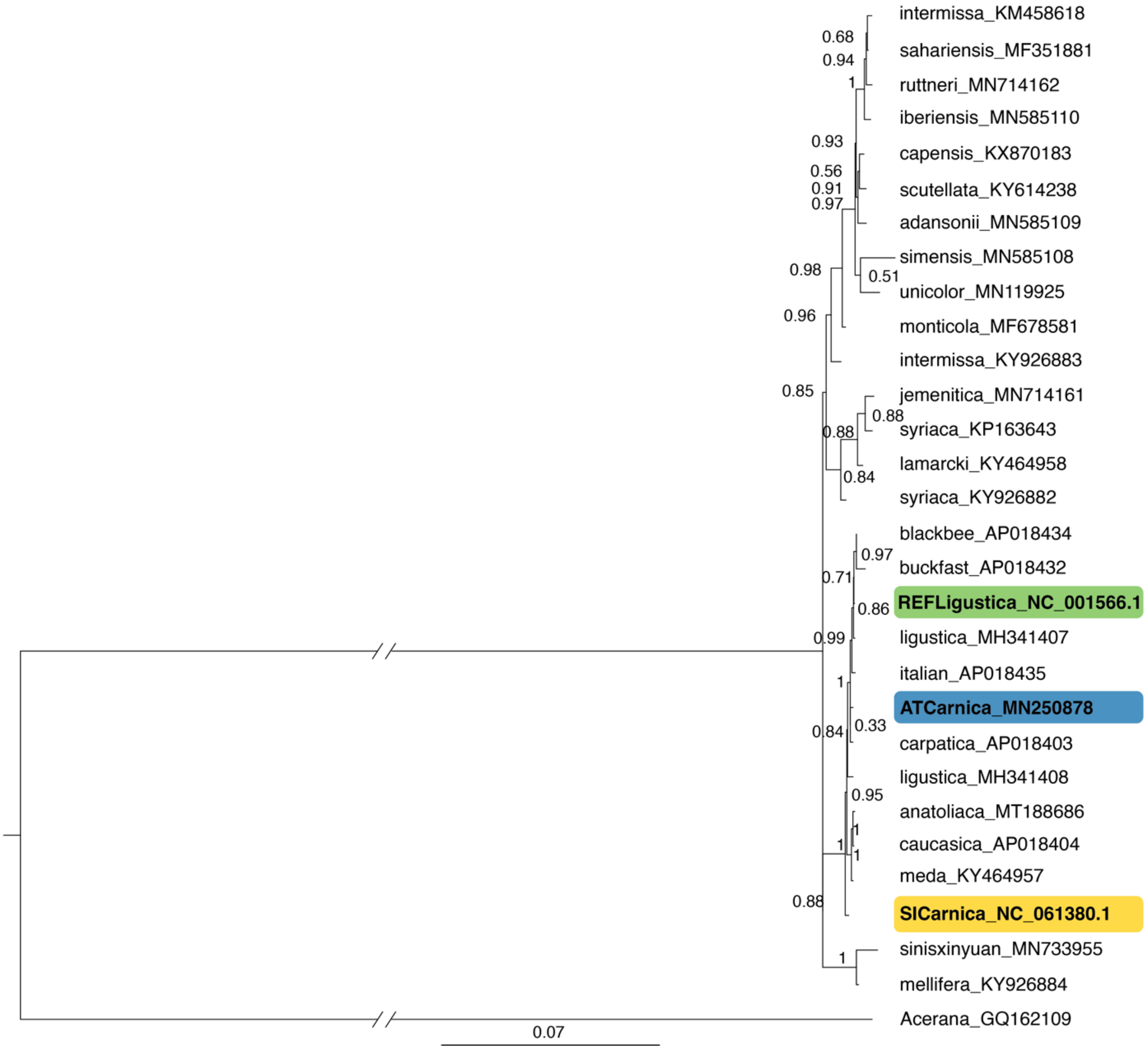

Figure S1. Maximum likelihood phylogenetic analysis of selected *A. mellifera* subspecies based on complete mitogenome sequences. Computed branch lengths are displayed. Bootstrap support values are presented on the nodes. Names of the samples include subspecies or strain name and GenBank accession number.
