# Supplementary material for: The Carniolan Honeybee from Slovenia—A Complete and Annotated Mitochondrial Genome with Comparisons to Closely Related Apis mellifera Subspecies
Source: Insects. 2022 Apr 22;13(5):403. doi: 10.3390/insects13050403 (PMC9146700; doi:10.3390/insects13050403)
Supplement: Supplementary file 1 [file insects-13-00403-s001.zip › insects-1411432-supplementary-Figure S3.pdf]

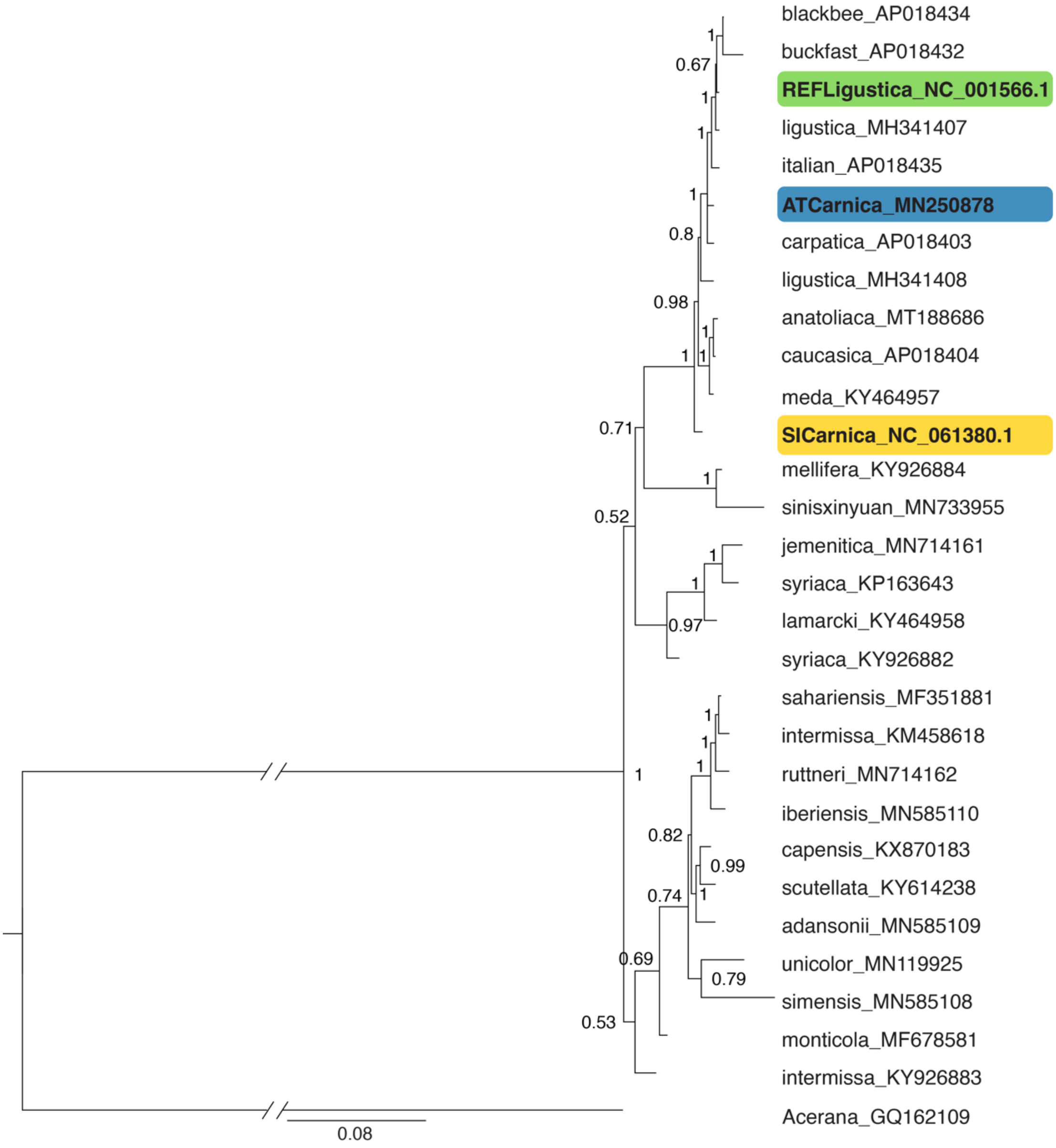

Figure S3. Bayesian phylogenetic analysis of selected *A. mellifera* subspecies using MrBayes software based on complete mitogenome sequences. Computed branch lengths are displayed. Posterior probabilities are presented on the nodes. Names of the samples include subspecies or strain name and GenBank accession number.
