# Supplementary material for: The Carniolan Honeybee from Slovenia—A Complete and Annotated Mitochondrial Genome with Comparisons to Closely Related Apis mellifera Subspecies
Source: Insects. 2022 Apr 22;13(5):403. doi: 10.3390/insects13050403 (PMC9146700; doi:10.3390/insects13050403)
Supplement: Supplementary file 1 [file insects-13-00403-s001.zip › insects-1411432-supplementary-Figure S5.pdf]

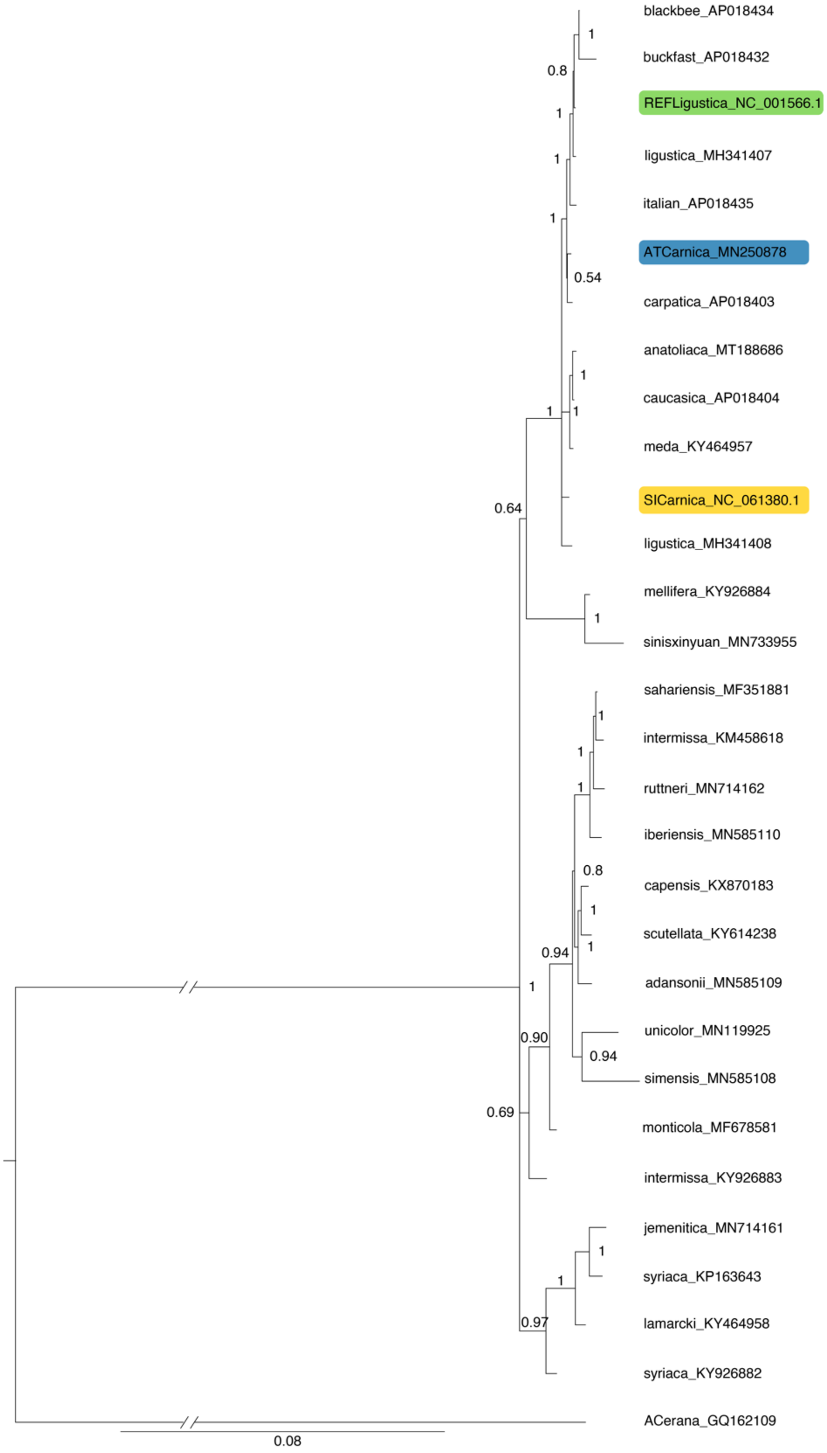

Figure S5. Bayesian phylogenetic analysis of selected *A. mellifera* subspecies using MrBayes software based on partial mitogenome sequences (GBLOCKS dataset). Computed branch lengths are displayed. Posterior probabilities are presented on the nodes. Names of the samples include subspecies or strain name and GenBank accession number.
