# Supplementary material for: The Carniolan Honeybee from Slovenia—A Complete and Annotated Mitochondrial Genome with Comparisons to Closely Related Apis mellifera Subspecies
Source: Insects. 2022 Apr 22;13(5):403. doi: 10.3390/insects13050403 (PMC9146700; doi:10.3390/insects13050403)
Supplement: Supplementary file 1 [file insects-13-00403-s001.zip › insects-1411432-supplementary-Figure S7.pdf]

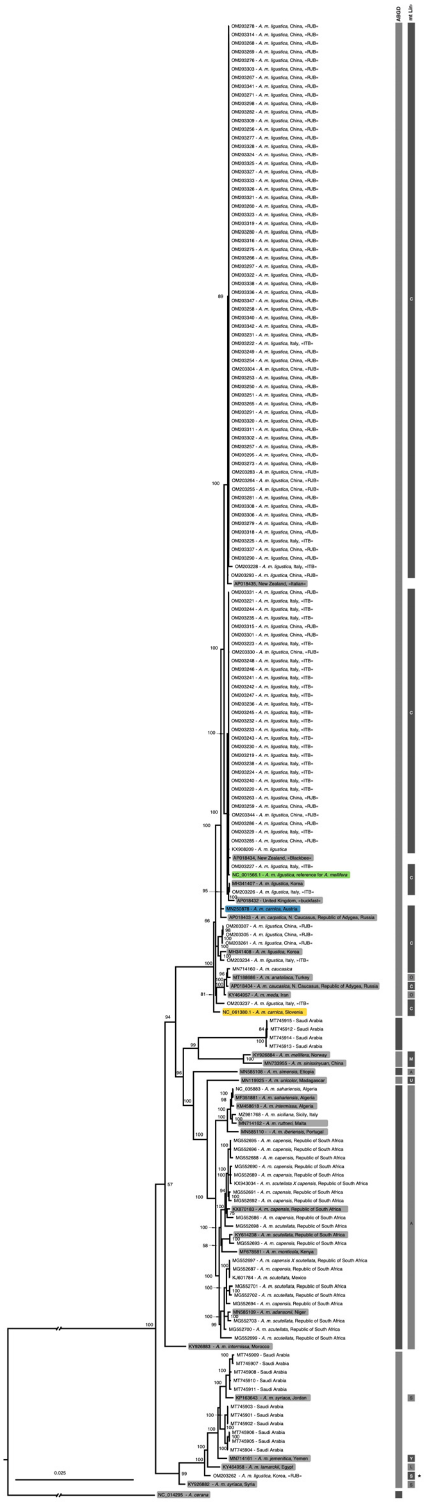

**Figure S7.** Bayesian phylogenetic analysis of extended *A. mellifera* subspecies using MrBayes software based on complete mitogenome sequences excluding control region. Computed branch lengths are displayed. Posterior probabilities are presented on the nodes. GenBank accession number, subspecies designation when applicable, geographic origin of the sample and strain in quotation marks are displayed on the leaves. Samples which were also included in other phylogenetic analyses are marked grey. SICarnica mitogenome is marked yellow. ATCarnica mitogenome is marked blue. REFLigustica mitogenome is marked green. Vertical grey lines specify the result of ABGD analysis, determining 8 separate groups. Each subspecies belonging to evolutionary lineage is

represented by the right-most vertical bars and letters M, C, O, S, L, Y, A and U (marked as mt Lineage). Lineage was assigned according to literature, when provided. * standing by sample OM203262 indicates a sample being determined as *A. m. ligustica* but belonging to S instead of C lineage, showing the discrepancies between subspecies and lineage designation when using only matrilineal information.
